# Knowledge and experience of Kazakhstan athletes in anti-doping and the impact of past educational intervention

**DOI:** 10.1186/s13011-022-00461-7

**Published:** 2022-04-26

**Authors:** Galiya Zhumabayeva, Gulnara Kapanova, Denis Vinnikov, Maira Bakasheva, Venera Abdulla, Andrej Grjibovski

**Affiliations:** 1 Al-Farabi Kazakh National University, Almaty, Kazakhstan; 2 Peoples’ Friendship, University of Russia, RUDN University), Moscow, Russian Federation; 3 Kazakhstan National Anti-Doping Center, Almaty, Kazakhstan; 4 Kazakhstan Medical University “Higher School of Public Health”, Almaty, Kazakhstan; 5 Northern State Medical University, Arkhangelsk, Russia; 6 I.M. Sechenov First Moscow State Medical University (Sechenov University), Moscow, Russia; 7 West Kazakhstan Marat Ospanov Medical University, Aktobe, Kazakhstan

**Keywords:** Kazakhstan, WADA, Anti-doping education, Doping, ALPHA test

## Abstract

**Background:**

Although Kazakhstan National Anti-Doping Organization (KazNADO) exists since 2013, but little is yet known about anti-doping (AD) knowledge of Kazakhstan athletes**.** The aim of this study was to assess the AD education knowledge level and experience among Kazakhstan athletes, as well as the impact of any past AD educational program on them.

**Methods:**

Altogether, 590 athletes (the median was age 17 years (interquartile range 8)), representing various sports, participated in the web-based study and completed the questionnaire, which consisted of socio-demographic part and ALPHA test. We assessed the association of any past AD education and experience with anti-doping knowledge using adjusted regression models.

**Results:**

A total of 54.6% participants underwent doping control and 82,7% of athletes received AD education at least once. More than 300 participants (50.8%) provided correct answers for 10 questions. Age and years in sports (competition duration) were significantly associated with the ALPHA scores of athletes. Athletes who received AD education more than once in the past had significantly higher ALPHA scores than non-AD educated athletes in most questions.

**Conclusion:**

AD education was associated with AD knowledge. Further research is needed to identify the adherence to anti-doping knowledge.

## Introduction

The use of prohibited substances to improve athletes’ performance is a pivotal issue in sports, and much evidence has now been accumulated that anti-doping rules violations (ADVR) are widely committed by the athletes of all levels (young, amateur and elite athletes) intentionally or non-intentionally [1–4]. The use of substances banned under the World Anti-Doping Code both in recreational and professional sport causes a big concern, associated with societal and public health consequences [5]. Doping scandals and doping prevalence statistics in various sports usually project anti-doping rules violations (ADRV) and prohibited substances usage by athletes of all levels [6]. Of note, previous studies demonstrated that around 10–15% of high-performance athletes reported the use of banned substances [7]. However, there may be some discordance between the study results and real-world situation.

Combatting doping in sports is important because of the increasing rate of use of prohibited substances and that of other associated antidoping rules violations, not just by elite and professional athletes, but also by amateurs and non-athletes [1]. This becomes a public health issue since doping use is associated with multiple health problems [8–10]. Furthermore, sports values in the society mold the associated social norms of prohibited substance use and vice versa [11, 12]. There exists sufficient evidence that social beliefs direct intentions and actual doping use patterns amongst athletes and non-athletes groups of various age.

A number of studies were focused only on secondary school athletes, where researchers attempted to find a link between knowledge about doping and tendencies to use it [2, 13, 14]. There are studies where the assessment of anti-doping knowledge was carried out during training camps or competitions, which could affect the test result [15]. Some studies were carried out within the framework of national programs (“High Five” (Germany), “100% Me” (Great Britain), “Be Fair, Play True” (Austria), “Mamma Parliamo di doping” (Italy), and “Cool and Clean” and “Real Winner” (Switzerland), the goals and objectives of these studies were to convey information about harmful effects of doping on children and adolescent athletes’ health, as well as on a decision-making process regarding doping.

Some of the studies listed above used PEAS (The Performance Enhancement Attitude Scale) questionnaire, WADA Play True test and self-created questionnaires [16–22]. Only Murofushi et al. used the ALPHA test to assess athletes’ anti-doping knowledge [23]. PEAS questions focus on doping behavior with psychological factors in an environmental context. Therefore, studies using PEAS are directed at attitudes and tendencies towards the use of doping, while the WADA ALPHA test assesses specific anti-doping knowledge. Questionnaires, which were created by the researchers themselves, were based on the objectives of the study, where it was also more determined how much knowledge could transfer into the behavior of an athlete and influence his adherence to clean sport [15, 16]. Most of the research on doping knowledge is aimed at the younger generation of athletes, due to the potential to influence the development of fair play values at a given age.

According to the literature, approximately 3–12% of young-age athletes used anabolic agents at a certain moment of their career [13, 14, 16, 24, 25]. From 2013 to 2020 in Kazakhstan, about 20% of all positive samples belonged to young athletes [26]. This alarming trend has forced Kazakhstan National Anti-Doping Organization (KazNADO) to improve and strengthen the existing anti-doping educational system, but the effect of these interventions has never been reported. Of note, there is a number of efficient educational programs aiming to avert the use of illegal performance-enhancing drugs, including the gender-specific U.S. college anti-doping programs ATLAS and ATHENA [14, 27, 28], the Swiss program Cool & Clean [17], programs in Iran [19], Sweden [18] and Japan [23].

In Kazakhstan, Ministry of Culture and Sports is financially and governmentally entitled to coordinate the sport system, which includes Olympic, Paralympic, Deaflympic, non-Olympic and National. There exist republican and regional specialized sports schools, sports federations and associations which are responsible for sport popularization and preparation of sport reserve for national teams.

The National Anti-Doping Organization (KazNADO) is a structural unit of the Ministry of Culture and Sports. KazNADO includes the education department, the doping control department, the scientific and methodological department and the administrative department. The work of the center includes educational activities, scientific and methodological support of the country's sports community, planning, doping control, managing the results of testing athletes, conducting investigations on anti-doping rule violations, international cooperation. While the Anti-Doping Activities Commissions are independent entities and exist outside the anti-doping center, but in close cooperation.

Educational activities include working with athletes, coaches, doctors and other athletes’ support personnel from Olympic and non-Olympic national teams on an ongoing basis. As an additional tool of educational activities, an anti-doping online course has been launched in Kazakh and Russian languages. Moreover, KazNADO has implemented anti-doping courses to specialized sports schools, Kazakh Academy of Sport and Tourism and other educational sports organizations.

The anti-doping education program in Kazakhstan has never been described, nor has the effect of this program on athletes. Therefore, we designed this study with the aim to assess the anti-doping education knowledge level among Kazakhstan athletes, and determine if the anti-doping education is associated with athletes’ education level with regard to anti-doping rules and regulations.

## Materials and methods

### Participants

The venue for this cross-sectional study were Kazakhstan Sport Federations and Specialized Sports Schools. Athletes of these sport entities resided in various cities of Kazakhstan, and the list of participating sport organizations were determined by the Education Plan of Kazakhstan National Anti-Doping Center. Requests for surveys occurred by agreement with multiple sports federations and specialized sports schools. All athletes regularly participated in sport competitions on different levels and thus were invited by their organizations.

Questionnaires were distributed among Kazakhstan athletes online due to Covid-19 pandemic (children, junior, international and elite). Participants (*N* = 590, 40% men) were recruited from November 2020 till May 2021. Athletes from over 10 sports participated in the study. Participants (*N* = 590, the median was age 17 years (interquartile range 8)) were 234 men (median age 20 years (interquartile range 11)) and 356 women (age 16 years (interquartile range 5)). Participating athletes represented various sports, such as athletics, gymnastics, weightlifting, shooting, archery, biathlon, canoe, water polo, swimming, etc. We obtained permission to conduct this study from the Ethics Committee of the Al-Farabi Kazakh National University, Almaty, Kazakhstan (IRB – A092). Participants were informed that their privacy would not be compromised and all signed an informed consent to participate online.

### Questionnaire

We prepared a structured survey in Russian and Kazakh. The tool was self-administered. It consisted of socio-demographic part, including sex, sport (athletics, gymnastics, weightlifting, shooting, archery, biathlon, canoe, water polo, swimming, etc.), ever-doping control experience (YES/NO), anti-doping (AD) education experience (no experience, once, or more than once), years in sports (competition duration) and highest level of competition (district, city, region, national, international level and Olympic and Paralympic Games level). Socio-demographic part was followed by the ALPHA test, (available at AD e-learning platform (ADeL), https://adel.wada-ama.org/learn), which measures AD knowledge level [20] and is available in a number of languages including Russian. This e-learning platform involved courses for athletes and athletes’ support personnel (coaches, doctors, administrators, parents) and anyone interested in the World Anti-Doping Code (Code) and clean sport.

ALPHA allows athletes to test their AD knowledge based on Code. There is no other suitable instrument to assess the AD education level of the Code, thus the ALPHA test was developed by World Anti-Doping Agency (WADA) scientists of social science research program, whereas its content validity was confirmed earlier. The test consisted of 12 questions with four answer options in each and only one correct answer. The ALPHA score was calculated by summing correct answers (score range 0–12).

This questionnaire was translated and adapted into Kazakh. The translation procedure consisted of direct translation by two independent translation agencies and a reverse translation by another two independent translation agencies. An anti-doping specialist then generated the final version of translated questionnaire. Direct translation from Kazakh to English and reverse translation were performed without intermediary translation to Russian.

### Statistical analysis

All variables were tested for normality with the Kolmogorov–Smirnov test and found not to be normally distributed. Therefore, we only used non-parametric tests in this analysis. The primary outcomes in this analysis were the n and percentage of subjects who provided correct answers to all ALPHA test questions. Variables were categorized in following way: age, age of first information about doping and duration of athlete’s career were treated as continuous variables. Sex was coded as ‘male’ and ‘female’. Competition level was categorized as ‘District’, ‘City’, ‘Region’, ‘National’, ‘International’, ‘Olympic Games’, and ‘Paralympic Games’. Educational level was coded as ‘Sport School and Sport college’ and ‘Higher education’. Anti-doping Education Experience for regression analysis was coded as 0 for ‘non-educated’ and 1 for ‘educated at least once’. Type of Anti-doping Education was coded as ‘not attended’, ‘lecture’, ‘online-course’, ‘Outreach’, ‘lecture + online-course’, ‘lecture + online course + outreach’, ‘lecture + outreach’, and ‘online course + outreach’. Doping Control Experience was coded as ‘No’, and ‘Yes’. Source of Information about Doping was coded as ‘none’, ‘coach’, ‘sport doctor’, ‘teammate’, ‘family’, ‘Internet’, ‘Anti-doping Education Program’. In addition to providing descriptive analysis of n and percent of correct answers reflecting knowledge and awareness, we also tested whether ever-doping control experience or anti-doping education affected these answers in the chi-squared tests.

We tested all twelve ALPHA questions whether they were associated with selected predictors, such as age, sex, competition level, competition duration, educational level, ever-anti-doping education and ever-doping control experience. Each of these predictors were first analyzed in a crude logistic regression, in which the outcome was a binary variable coded as 0 or 1 for a wrong and correct answer. Variables that were significantly associated with the outcomes in crude analyses were then included in the multivariable models. Following crude models, we then adjusted each model for confounders, including all other predictors from the selected list. Confounders were chosen based on the bivariate models. In all regression models, we report the odds ratios (OR) with their corresponding 95% confidence intervals (CI). Tables with bivariate comparisons report medians with their interquartile ranges (IQR) or n with percent for the group. All statistical processing was performed with SPSS Statistics 26.0 (IBM, USA).

## Results

Most responders were from Sport Schools and Colleges with secondary general education level of education (*n* = 380 (64.4%)). Of all, 277 (46.9%) of participants received information about doping from Internet, 160 (27.1%) from their coach and 70 (11.9%) from sport doctors. One hundred and two responders (17.3%) have never experienced any type of anti-doping education, whereas 488 (82.7%) were exposed to at least one anti-doping activity. Anti-doping education included not only one anti-doping education activity, but also some other options (Table 1).

**Table 1:** Socio-demographic characteristics of the study participants (*N* = 590)

Variable	n (%)
Sex	
Male	234 (40)
Female	356 (60)
Age, years	
Median (IQR)	17 (8)
Range	15–39
Sports	
Athletics	119 (20.3)
Rhythmic Gymnastics	108 (18.3)
Acrobatic gymnastics	64 (10.8)
Artistic gymnastics	44 (7.5)
Weightlifting	41 (6.9)
Shooting	29 (4.9)
Archery	19 (3.2)
Biathlon	18 (3.1)
Canoe	14 (2.4)
Water Polo	13 (2.2)
Swimming	12 (2.0)
Other	109 (18.4)
Competition Duration, years	
Mean ± SD	7.5 ± 5
Range	1–25
Competition Level	
District	4 (0.7)
City	13 (2.2)
Region	32 (5.4)
National	166 (28.1)
International	328 (55.6)
Olympic Games	44 (7.5)
Paralympic Games	3 (0.5)
Education Level	
Sport School	297 (50.3)
Sport College	83 (14.1)
Bachelor Degree in Sport	171 (29.0)
Master Degree in Sport	32 (5.4)
PhD in Sport	7 (1.2)
Age of First Information about Doping	
7–17 years	481 (81.6)
18–22 years	87 (14.7)
≥ 23 years	22 (3.7)
Primary source of Information about Doping	
None	6 (1.0)
Coach	160 (27.1)
Sport Doctor	70 (11.9)
Teammate	42 (7.1)
Family	18 (3.1)
Internet	277 (46.9)
Anti-doping Education Program	17 (2.9)
Doping Control Experience	
Yes	322 (54.6)
No	268 (45.4)
Anti-doping Education Experience	
Non-educated	102 (17.3)
Once	231 (39.2)
More than once	257 (43.5)
Type of Anti-doping Education	
Not attended	102 (17.3)
Lecture	130 (22.0)
Online-course	115 (19.5)
Outreach	6 (1.0)
Lecture + Online course	158 (26.8)
Lecture + Online course + Outreach	58 (9.8)
Lecture + Outreach	11 (1.9)
Online course + Outreach	10 (1.7)

*Data are presented as medians with the corresponding interquartile range (IQR) or n (%)*

Table 2 shows the overall percent of correct answers to ALPHA test questions. We received more than 300 correct answers for 10 questions. We found a very wide range of correct answers, indicative of a significant magnitude in knowledge level among the athletes. With almost 100% awareness on the conditions allowing to refuse from the test (95.1%), philosophy behind doping control remained poorly understood by the athletes (only 24.2% correct answers). The prohibited list itself was familiar for almost 83% participants.

**Table 2:** Number of correct ALPHA answers per question

ALPHA questionnaire		All (*N* = 590)	
No	Questions	Number of correct answers (n)	(%)
1	How can an athlete with a medical condition decide whether to take a medication?	411	69.7
2	What are the athlete’s rights when a positive test is returned?	334	56.6
3	What are the side effects of using anabolic steroids?	348	59.0
4	What condition allows an athlete to refuse to be tested?	561	95.1
5	What does TUE stand for?	436	73.9
6	What is the philosophy behind anti-doping?	143	24.2
7	What is the Prohibited List?	488	82.7
8	What is the purpose of the World Anti-Doping Code?	296	50.2
9	What is the requirement for laboratories that analyze blood or urine samples for doping control?	433	73.4
10	When do athletes have to tell their National Anti-Doping Organization where they will be living, training and competing?	386	65.4
11	When must an athlete be notified of an upcoming test?	338	57.3
12	Who is responsible for the substances found in an athlete’s body?	445	75.4

Table 3 shows that some doping control experience had a positive impact on the knowledge level. Thus, it was associated with more correct answers in seven questions out of twelve. Of note, such experience did not affect the least known question of the philosophy behind doping. We also found significant differences in awareness level on most questions with regard to ever-education. “More than once education” resulted in significantly better knowledge in 11 out of 12 questions, even with regard to the least known question on the philosophy (Table 3). Further analysis did not show any statistically significant association between the types of Anti-Doping Education and ALPHA answers.

**Table 3:** Comparison of ALPHA scores between doping control experience and anti-doping education experience (*N* = 590)

ALPHA questionnaire	Number of correct answers n (%)	Doping Control Experience n (%)			Anti-doping Education n (%)			
		Yes	No	*p* value	Non-educated	Once	More than once	*p* value
1. How can an athlete with a medical condition decide whether to take a medication?	411 (69.7)	239 (74.2)	172 (64.2)	*p* = 0.008	38 (37.3)	158 (68.4)	215 (83.7)	*p* < 0.001
2. What are the athlete’s rights when a positive test is returned?	334 (56.6)	183 (56.3)	151 (56.3)	*p* = 0.905	57 (55.9)	124 (53.7)	153 (59.5)	*p* = 0.422
3. What are the side effects of using anabolic steroids?	348 (59.0)	191 (59.3)	157 (58.6)	*p* = 0.857	46 (45.1)	117 (50.6)	185 (72.0)	*p* < 0.001
4. What condition allows an athlete to refuse to be tested?	561 (95.1)	315 (97.8)	246 (91.8)	*p* = 0.001	88 (86.3)	220 (95.2)	253 (98.4)	*p* < 0.001
5. What does TUE stand for?	436 (73.9)	256 (79.5)	180 (67.2)	*p* = 0.001	52 (51.0)	173 (74.9)	211 (82.1)	*p* < 0.001
6. What is the philosophy behind anti-doping?	143 (24.2)	86 (26.7)	57 (21.3)	*p* = 0.125	15 (14.7)	61 (26.4)	67 (26.1)	*p* = 0.047
7. What is the Prohibited List?	488 (82.7)	277 (86.0)	211 (78.7)	*p* = 0.020	68 (66.7)	193 (83.5)	227 (88.3)	*p* < 0.001
8. What is the purpose of the World Anti-Doping Code?	296 (50.2)	169 (52.5)	127 (47.4)	*p* = 0.218	39 (38.2)	115 (49.8)	142 (55.3)	*p* = 0.014
9. What is the requirement for laboratories that analyze blood or urine samples for doping control?	433 (73.4)	270 (83.9)	163 (60.8)	*p* < 0.001	47 (46.1)	159 (68.8)	227 (88.3)	*p* < 0.001
10. When do athletes have to tell their National Anti-Doping Organization where they will be living, training and competing?	386 (65.4)	230 (71.4)	156 (58.2)	*p* = 0.001	53 (52.0)	132 (57.1)	201 (78.2)	*p* < 0.001
11. When must an athlete be notified of an upcoming test?	338 (57.3)	205 (63.7)	133 (49.6)	*p* = 0.001	46 (45.1)	109 (47.2)	183 (71.2)	*p* < 0.001
12. Who is responsible for the substances found in an athlete’s body?	445 (75.4)	250 (77.6)	195 (72.8)	*p* = 0.171	59 (57.8)	171 (74.0)	215 (83.7)	*p* < 0.001

*Chi-squared test was used for calculations*

We analyzed all twelve questions from the ALPHA test with regard to their association with predictors in logistic regression models. Table 4 presents the associations of selected predictors, such as age, competition level, years in competition, educational level, ever-anti-doping education and ever-doping control experience, with the correct answers. Educational level was not associated with correct answers in any question (Table 4). Competition level in adjusted models was associated with correct answers on six questions, indicative of better knowledge with advanced level of training, but not with the remaining six questions. Years in competition and doping control experience could positively predict better knowledge level on question 12 and question 9, respectively. Although included in all models, sex demonstrated statistically significant association with question 6 (OR 0.49; 95% CI 0.33–0.73) only. The most pronounced was the effect of anti-doping education. Thus, such education increased the likelihood of good knowledge in all questions except questions 2, 10 and 11 (Table 4).

**Table 4:** Adjusted odds ratios with 95% confidence intervals in multivariable logistic regression models (*N* = 590)

ALPHA questions	Number of correct answers (%)	Predictors					
		**Age**	**Competition Level**	**Competition Duration**	**Education Level**	**Anti-doping Education**	**Doping Control Experience**
**No. 1**	411 (69.7)		1.30 (1.02–1.65)^a^	0.99 (0.95–1.04)		4.82 (2.99–7.76)^a^	1.04 (0.69–1.57)
**No. 2**	334 (56.6)	1.01 (0.97–1.05		1.05 (0.99–1.11)			
**No. 3**	348 (59.0)	1.11 (1.06–1.17)^a^	1.25 (1.01–1.55)^a^		0.67 (0.39–1.16)	1.62 (1.03–2.55)^a^	
**No. 4**	561 (95.1)	1.13 (0.96–1.33)		0.97 (0.83–1.13)	1.10 (0.24–5.04)	3.77 (1.68–8.49)^a^	2.18 (0.85–5.64)
**No. 5**	436 (73.9)					2.95 (1.85–4.72)^a^	1.47 (0.97–2.25)
**No. 6**	143 (24.2)					2.03 (1.11–3.71)^a^	
**No. 7**	488 (82.7)	1.09 (1.00–1.19)^a^	1.51 (1.15–1.99)^a^	0.97 (0.89–1.06)	2.04 (0.89–4.72)	2.53 (1.47–4.34)^a^	0.83 (0.50–1.35)
**No. 8**	296 (50.2)	1.04 (1.00–1.09)^a^	1.19 (0.96–1.48)	0.99 (0.93–1.04)		1.59 (1.01–2.50)^a^	
**No. 9**	433 (73.4)	1.15 (1.06–1.24)^a^	1.34 (1.04–1.72)^a^	0.98 (0.90–1.05)	0.89 (0.44–1.80)	3.25 (1.98–5.33)^a^	1.87 (1.22–2.88)^a^
**No. 10**	386 (65.4)	1.07 (1.01–1.14)^a^	1.38 (1.09–1.74)^a^	1.00 (0.94–1.07)	1.49 (0.83–2.67)	1.50 (0.93–2.41)	1.05 (0.71–1.55)
**No. 11**	338 (57.3)	1.09 (1.03–1.15)^a^	1.52 (1.20–1.92)^a^	0.99 (0.93–1.05)	1.63 (0.95–2.80)	1.34 (0.83–2.16)	0.99 (0.68–1.461)
**No. 12**	445 (75.4)	0.97 (0.92–1.03)	1.21 (0.94–1.54)	1.10 (1.03–1.18)^a^	1.63 (0.86–3.08)	2.16 (1.35–3.47)^a^	

^a^Statistically significant

Note: Variables not associated with the outcomes in bivariate analyses were not included in the multivariable models. All models were adjusted for age, competition level, competition duration, education level, anti-doping education, doping control experience. No.1—How can an athlete with a medical condition decide whether to take a medication? No.2—What are the athlete’s rights when a positive test is returned? No.3—What are the side effects of using anabolic steroids? No.4—What condition allows an athlete to refuse to be tested? No.5—What does TUE stand for? No.6—What is the philosophy behind anti-doping? No.7—What is the Prohibited List? No.8—What is the purpose of the World Anti-Doping Code? No.9—What is the requirement for laboratories that analyze blood or urine samples for doping control? No.10—When do athletes have to tell their National Anti-Doping Organization where they will be living, training and competing? No.11—When must an athlete be notified of an upcoming test? No.12—Who is responsible for the substances found in an athlete’s body?”.

## Discussion

This is the first study, conducted on a large sample of 590 Kazakhstan athletes, reporting awareness level of these athletes on doping, anti-doping regulations, past experience with doping control and the impact of past anti-doping education on the awareness level. In the current analysis, the most pronounced was the effect of anti-doping education, which increased the likelihood of good knowledge 2–fourfold.

The questions which scored a small number of correct answers were “What is the philosophy behind anti-doping?” and “What is the purpose of the World Anti-Doping Code?”. The greatest number of correct answers (561 athletes) were related to the question: “What condition allows an athlete to refuse to be tested?”. This may highlight that for athletes information about doping control is more important. We found that anti-doping education experience in athletes’ background was associated with the number of correct answers to all questions, except “What are the athlete’s rights when a positive test is returned?”, “When do athletes have to tell their National Anti-Doping Organization where they will be living, training and competing?” and “When must an athlete be notified of an upcoming test?”. Irrelevance of question 2 for most athletes implies poor awareness of the procedure following the notification on this anti-doping rule violation. Questions 10 and 11 require higher level of anti-doping knowledge, usually consistent with higher athletes’ performance level. Further regression analysis conducted for all ALPHA questions with the anti-doping education program (as a source of information about Doping), age, and competition duration as predictors, showed statistical significance only in the question No.1 (“How can an athlete with a medical condition decide whether to take a medication?”) and question No.2 (“What are the athlete’s right when a positive test is returned?”). These two questions likely reflected the most important information for the athletes. And such predictors as anti-doping education program as a source of information about doping, age and competition duration affected the number of correct answers to questions 1 and 2. The analysis did not reveal statistical significance with the above-mentioned predictors in the remaining ten questions. Therefore, we concluded that they were not of practical importance for the athletes.

Murofushi et al., who used the same ALPHA questionnaire in their study, set strict sample selection criteria, which included only students from sport university [23]. This implies a biased result, since the sports universities include anti-doping programs or information about doping as part of their curriculum. However, the Japanese study also revealed a statistically significant association between anti-doping education and athletes’ anti-doping knowledge, when both the experience of doping control and the anti-doping knowledge of athletes did not show statistical significance.

Studies on attitudes towards doping and beliefs have identified other factors influencing athletes’ decision regarding the use of prohibited substances, including but not limited to socio-economic conditions, entourage, parents’ beliefs and expectations, teammates, and Internet, directing further research in Kazakhstan [20–22].

There are some limitations of our study that need to be addressed. Firstly, cross-sectional design of this study did now allow checking and ascertaining causality in the associations we had identified. Secondly, we only conducted the test once, we did not make any intervention as anti-doping seminars or lecture prior and after knowledge assessment, so have not monitored the progress in athletes’ knowledge. Thirdly, we have taken a wide range of age, competition experience and education level, which might affect the responses and results. The use of 0.05 threshold for significance testing taking into account the absence of control over the Type I error rate we consider as the fourth limitation of our study.

### Implications

Our findings exhibit some effect of the existing Kazakhstan anti-doping program, in which athletes, who have undergone anti-doping education courses and doping control, showed higher level of AD knowledge. Therefore, the present study has preliminary implications to introduce anti-doping programs not only to sports environment, but also to the educational system of the universities and general education schools to prevent the use of prohibited and dangerous substances by Kazakhstan athletes in future. Moreover, efficient and practical anti-doping policy should be widely implemented to the national sports system. These results highlight the areas, where a systematic approach should be used to improve the level of anti-doping knowledge among athletes in order to retain sport as a health-enhancing occupation.

## Conclusions

In conclusion, this is the first analysis from Central Asian countries describing the level of anti-doping knowledge in the sport population. In this study the usefulness of anti-doping education and the need for educational interventions were found. Continuous analysis is crucial to fully identify the factors affecting the prevalence of doping in Kazakhstan sport. Further research is needed to identify Kazakhstani athletes’ attitudes towards doping use in the framework of existing national anti-doping education system.

## Data Availability

The datasets generated and analyzed during the current study are available from the corresponding author on reasonable request.
